# Trajectories and determinants of ageing in Portugal: insights from EpiDoC, a nationwide population-based cohort

**DOI:** 10.1186/s12889-023-16370-8

**Published:** 2023-08-17

**Authors:** David G. Lopes, Nuno Mendonça, Ana Rita Henriques, Jaime Branco, Helena Canhão, Ana M Rodrigues

**Affiliations:** 1 EpiDoC Unit, NOVA Medical School, CHRC, Universidade NOVA de Lisboa, Rua do Instituto Bacteriológico, nº5, Lisboa, 1150-082 Portugal; 2 Centro Hospitalar Lisboa Ocidental (CHLO-E.P.E.), Serviço de Reumatologia do Hospital Egas Moniz, Lisboa, Portugal; 3 Rheumatology Unit, Hospital dos Lusíadas, Lisboa, Portugal

**Keywords:** Disability, Health-related quality of life, Older adults, Trajectories, Longitudinal

## Abstract

**Introduction:**

The population in Portugal is ageing due to increased life expectancy and reduced fertility rates. We aimed to estimate the health trajectories of Portuguese older adults (60 + years old) in a 10-year period and to assess associated sociodemographic, lifestyle factors and multimorbidity status.

**Methods:**

Using the population-based EpiDoC cohort, we estimated the trajectories of health-related quality of life and physical function of 4135 Portuguese older adults over 10 years using linear mixed models. Factors associated to health-related quality of life and physical function were assessed using linear mixed models and random intercept tobit regression, respectively.

**Results:**

The physical disability of participants increased by 0.263 (0.240, 0.286), and health-related quality of life declined by 0.074 (-0.084, -0.063), over 10 years. With advancing age, older adults reported a faster reduction in health-related quality of life and faster increase in physical disability. In general, women were in worse health than men at baseline, albeit with a similar rate of change throughout the follow-up. Higher education and regular exercise were associated with better health-related quality of life and physical function while multimorbidity and excess weight were associated with worse reporting of these outcomes.

**Conclusions:**

These findings, based on longitudinal data with 10 years of follow-up, are essential to effectively plan resource allocation, plan better healthcare and design informed public health policies in Portugal.

**Brief summary:**

This study characterizes ageing in Portugal showing increased physical disability and decreased health-related quality of life with advancing age older adults, helping to develop public health policies.

**Supplementary Information:**

The online version contains supplementary material available at 10.1186/s12889-023-16370-8.

## Key points

• Increased life expectancy and lower fertility rates are causing the Portuguese population to age rapidly.

• The results of this long-term follow-up study show increased physical disability and decreased health-related quality of life with advancing age in older adults, and a faster worsening of these outcomes at older ages. Older adults with higher education and regular exercise had better health-related quality of life and physical function while multimorbidity and excess weight were associated with worse reporting of these outcomes.

• This study characterizes ageing in Portugal which is essential to develop evidence-based public health policies.

**Supplementary Information:**

The online version contains supplementary material available at 10.1186/s12889-023-16370-8.

## Introduction

In 2021, older adults (60 + years) comprised 27.4% of Europeans, an increase of 3.6% from 23.8% a decade earlier [1]. Portugal has one of the oldest populations in Europe due to an increase in life expectancy and reduced fertility rates. In that same year, older adults comprised 29.1% of the population, an increase of 4.5% from 24.6% 10 years before [1]. Regarding general life expectancy in Portugal, a person with 60 + years in 2010 should expect to live on average 23.1 years, and in 2019 that average expectancy increased to 24.3 years [2]. However, healthy life expectancy (HALE) in Portugal, the number of years an individual can expect to live in good health or disability-free, has not kept pace. HALE at age 60 was estimated to be 16.3 years for men and 18.8 years for women in 2010, and 17.3 years for men and 19.8 years for women in 2019 [2]. These estimates are considerably lower than the European Union’s (EU) average and the gap has been widening [2].

The increase in the number of older adults, years spent in bad health and lower fertility rates present new challenges to Portuguese governments. To this end, having up to date and robust estimates of the ageing trajectories of Portuguese older adults is essential for decision-makers to design informed public health policies, and effectively plan budgetary and human resource allocation to long-term care and healthcare, targeting those that need it the most, as well as ensuring availability of carers and supporting informal carers. However, there is a scarcity of population-based health data in Portugal, especially for older adults and that prospectively follows individuals for extended periods of time. We previously showed that Portuguese older adults had a high prevalence of multimorbidity (78%) and unhealthy lifestyles which further increase the vulnerability of this group and the need for dedicated interventions [3].

Following from previous results and to provide a more informed picture of the health of Portuguese older adults (60 + years old), we aimed to estimate the trajectories of older men and women, over 10 years in relation to health-related quality of life and physical function as well as to assess associated sociodemographic, lifestyle and health factors.

## Methods

### Study population

**Fig. 1:**
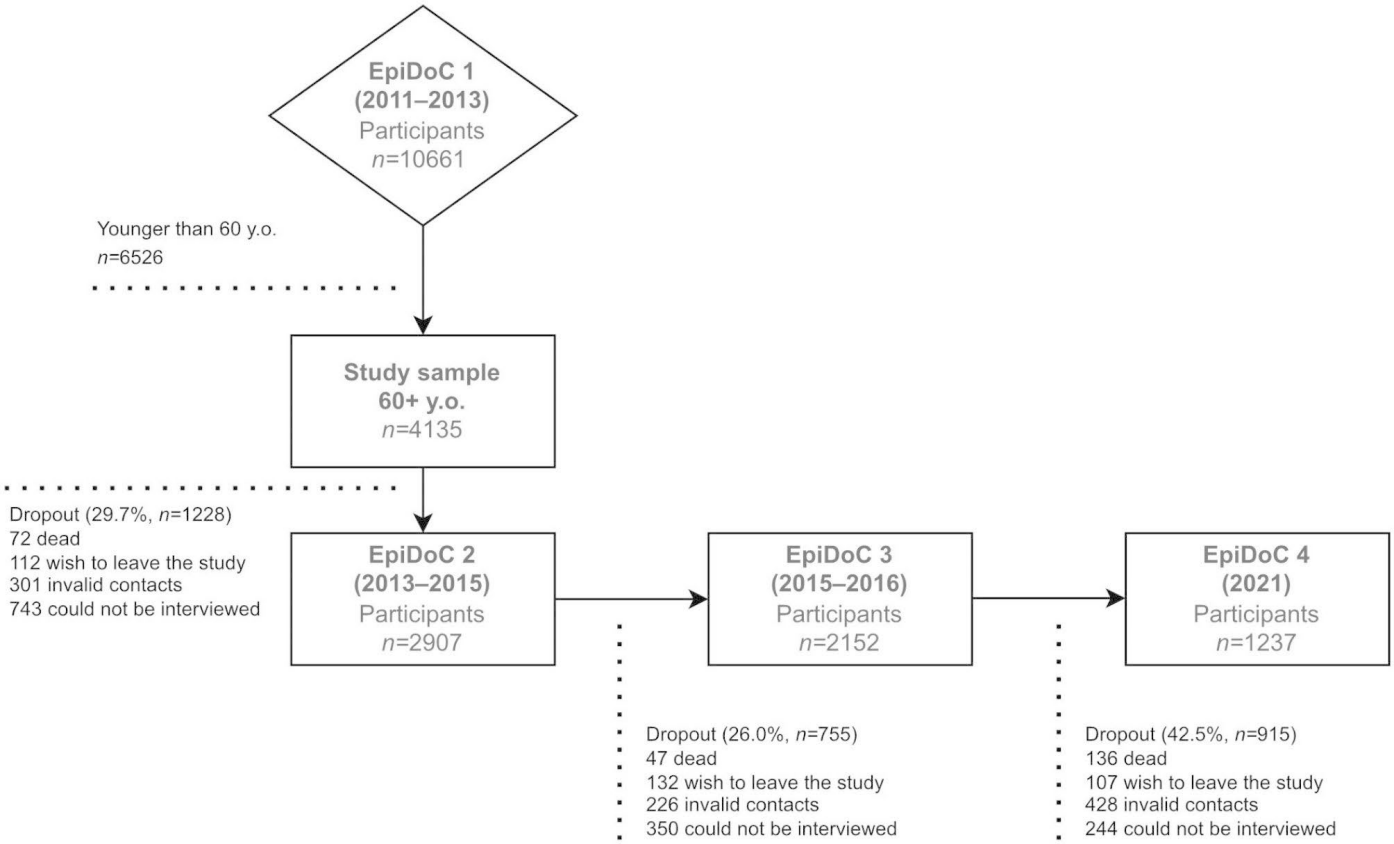
EpiDoC cohort’s profile for participants 60 years or older at baseline

The EpiDoC cohort is composed of a nationally representative sample of the adult Portuguese population (≥ 18 years old) living in private households of mainland Portugal and Islands (Madeira and Azores). Details on recruitment and sampling are described elsewhere [4]. Briefly, at baseline (wave 1 - September 2011 to December 2013), 10,661 participants were visited in their homes by a team of trained research assistants where a structured questionnaire on sociodemographics, lifestyles and health was used. Subsequent follow-up waves (wave 2 – March 2013 to July 2015, wave 3 – September 2015 to July 2016, wave 4 – March to August 2021) were conducted through phone call interviews by trained research assistants, using the same core questionnaire. This study included 4135 participants aged 60 or older at baseline (EpiDoC 1) that were followed throughout subsequent waves, i.e., EpiDoC 2 (*n =* 2907), EpiDoC 3 (*n =* 2152) and the final wave, EpiDoC 4 (*n =* 1237). The most common reasons for non-participation in follow-up waves were being unable to interview the eligible participant, invalid contacts, wishing to leave the study, or death (Fig. 1).

### Physical function (disability index and domains)

Physical function was assessed using the European-Portuguese version of the Health Assessment Questionnaire (HAQ) that evaluates the difficulty performing 20 common daily activities over the previous week with four possible answers each (without difficulty, some difficulty, with much difficulty, unable to do). These were classified into 8 domains: dressing and grooming; arising; eating; walking; hygiene; reach; grip; and common daily activities (e.g., running errands and shopping, getting in and out of a car and doing chores). A disability index was computed for HAQ ranging from 0 (no disabilities) to 3 (complete disability) [5]. A higher disability index can be interpreted as less functional ability.

### Health-related quality of life

Health-related quality of life (HRQoL) was measured using the European-Portuguese validated version of the EuroQol 5-dimensions questionnaire (EQ-5D), comprised of five dimensions (mobility, self-care, usual activities, pain/discomfort, and anxiety/depression) with three possible responses each (without problems, some problems, extreme problems). The descriptive system was converted into a summary index score ranging from − 1 (states worse than death, with 0 equivalent to death) to 1 (full health) [6]. A lower EQ-5D score can be interpreted as lower HRQoL.

### Health and lifestyle variables

Self-reported chronic non-communicable diseases at baseline (wave 1), and at wave 3 and wave 4 were assessed through the question: “Has a doctor ever told you that you suffer from the following chronic diseases?”. At wave 2 it was assessed through the report of new diagnosis since the previous contact. Variables were cleaned and harmonized across waves, and multimorbidity was defined as self-reporting two or more diseases from the following list of chronic non-communicable diseases: high blood pressure, high cholesterol, cardiac disease, diabetes mellitus, chronic lung disease, problems in the digestive tract, neurological disease, mental disease, cancer, and hyperuricemia.

Regular exercise was assessed in all waves through the question “Do you practice regular exercise/sports (yes, no, doesn’t know/doesn’t answer)” and the frequency of exercise per week considered as the number of days of regular exercise per week. Regular exercise was then recategorized into three strata: no regular exercise, frequent (1 or 2 times per week) or very frequent (at least 3 times per week). Frequencies of exercise reported as occasionally or rarely were excluded.

Self-reported weight and height were used to calculate body mass index (BMI) (weight in kg/height in m2) which was categorized as underweight (< 18.5 kg/m2), Normal weight (18.5-24.99 kg/m2), overweight (25-29.99 kg/m2) and obese (≥ 30 kg/m2). Other lifestyle variables included smoking habits (“never”, “in the past”, “active smokers”) and alcohol drinking as “no” and “yes” (recoded from occasional or daily alcohol consumption).

### Sociodemographic variables

Sociodemographic variables were collected at baseline: sex, age, NUTS II region (Lisbon, North, Centre, Algarve, Alentejo, Madeira and Azores), marital status, and education level. Marital status was categorized as “single”, “married or partnership”, “divorced” and “widow”. Education level was categorized as: “<4 years” (unfinished 1st cycle/primary education), “4–9 years” (finished primary education/1st cycle or 2nd or 3rd cycle), “10–12 years” (secondary education) and “>12 years” (university education).

### Statistical analysis

Baseline participant characteristics are shown as frequency (percentage) for categorical variables and mean (standard deviation) for continuous variables. Participants were described by baseline age group (Table 1) and by sex (e-Table 1). All variables were cleaned and harmonized prior to restructuring for longitudinal analysis.

**Table 1:** Baseline sociodemographic and lifestyle characteristics of older adults from the EpiDoC cohort by age group

	All *n =* 4135	60-64y *n =* 966	65-69y *n =* 943	70-74y *n =* 819	75-79y *n =* 737	80-84y *n =* 437	85 + y *n =* 233
**Sociodemographic**							
**Sex**							
Men	1510 (36.5%)	372 (38.5%)	359 (38.1%)	300 (36.6%)	260 (35.3%)	140 (32.0%)	79 (33.9%)
Women	2625 (63.5%)	594 (61.5%)	584 (61.9%)	519 (63.4%)	477 (64.7%)	297 (68.0%)	154 (66.1%)
**Age** (years)							
Mean (SD)	71.7 (7.8)	62.4 (1.5)	67.4 (1.5)	72.3 (1.4)	77.4 (1.4)	82.1 (1.5)	88.7 (3.7)
**Region** (NUTS II)							
North	1168 (28.2%)	286 (29.6%)	283 (30.0%)	227 (27.7%)	206 (28.0%)	112 (25.6%)	54 (23.2%)
Centre	898 (21.7%)	201 (20.8%)	166 (17.6%)	192 (23.4%)	182 (24.7%)	102 (23.3%)	55 (23.6%)
Lisbon	869 (21.0%)	202 (20.9%)	216 (22.9%)	168 (20.5%)	142 (19.3%)	93 (21.3%)	48 (20.6%)
Alentejo	319 (7.7%)	64 (6.5%)	72 (7.6%)	53 (6.5%)	70 (9.5%)	35 (8.0%)	26 (11.2%)
Algarve	165 (4.0%)	32 (3.3%)	31 (3.3%)	39 (4.8%)	31 (4.2%)	21 (4.8%)	11 (4.7%)
Azores	328 (7.9%)	83 (8.6%)	91 (9.7%)	51 (6.2%)	52 (7.1%)	31 (7.1%)	20 (8.6%)
Madeira	388 (9.4%)	99 (10.2%)	84 (8.9%)	89 (10.9%)	54 (7.3%)	43 (9.8%)	19 (8.2%)
**Marital status**							
Single	190 (4.6%)	57 (5.9%)	48 (5.1%)	30 (3.7%)	16 (2.2%)	25 (5.7%)	14 (6.0%)
Married/Partnership	2491 (60.3%)	686 (71.1%)	620 (65.9%)	539 (65.8%)	396 (53.7%)	178 (40.7%)	72 (30.9%)
Divorced	211 (5.1%)	89 (9.2%)	65 (6.9%)	18 (2.2%)	21 (2.8%)	13 (3.0%)	5 (2.1%)
Widowed	1240 (30.0%)	133 (13.8%)	208 (22.1%)	232 (28.3%)	304 (41.2%)	221 (50.6%)	142 (60.9%)
**Education level**							
< 4 years	1201 (29.2%)	83 (8.6%)	198 (21.2%)	252 (31.0%)	330 (44.9%)	206 (47.7%)	132 (56.9%)
4–9 years	2460 (59.8%)	719 (74.5%)	628 (66.9%)	485 (59.7%)	354 (48.2%)	197 (45.6%)	77 (33.2%)
10–12 years	229 (5.6%)	92 (9.5%)	49 (5.2%)	41 (5.0%)	25 (3.4%)	11 (2.5%)	11 (4.7%)
> 12 years	226 (5.5%)	71 (7.4%)	64 (6.8%)	35 (4.3%)	26 (3.5%)	18 (4.2%)	12 (5.2%)
**Lifestyle**							
**Body mass index** (kg/m^2^)							
Mean (SD)	27.6 (4.6)	27.9 (4.9)	28.1 (4.7)	27.7 (4.5)	27.3 (4.1)	26.7 (4.3)	25.4 (4.1)
Underweight (< 18.5)	38 (1.0%)	13 (1.4%)	4 (0.4%)	8 (1.1%)	2 (0.3%)	7 (1.9%)	4 (2.4%)
Normal weight (18.5–24.9)	1114 (29.8%)	247 (26.8%)	236 (26.2%)	218 (29.3%)	190 (29.5%)	139 (38.5%)	84 (50.6%)
Overweight (25-29.9)	1589 (42.5%)	395 (42.9%)	397 (44.1%)	313 (42.1%)	296 (46.0%)	132 (36.6%)	56 (33.7%)
Obese (≥ 30)	994 (26.6%)	265 (28.8%)	263 (29.2%)	205 (27.6%)	156 (24.2%)	83 (23.0%)	22 (13.3%)
**Smoking habits**							
Never	2980 (72.2%)	610 (63.2%)	667 (70.7%)	604 (73.8%)	571 (77.5%)	339 (77.9%)	189 (81.5%)
In the past	878 (21.3%)	229 (23.7%)	208 (22.1%)	175 (21.4%)	142 (19.3%)	86 (19.8%)	38 (16.4%)
Active smokers	272 (6.6%)	126 (13.1%)	68 (7.2%)	39 (4.8%)	24 (3.3%)	10 (2.3%)	5 (2.2%)
**Alcohol drinking**							
No	2803 (50.4%)	431 (44.7%)	438 (46.4%)	409 (49.9%)	407 (55.2%)	253 (58.2%)	145 (62.8%)
Yes	2046 (49.6%)	533 (55.3%)	505 (53.6%)	410 (50.1%)	330 (44.8%)	182 (41.8%)	86 (37.2%)

All values are n (%) unless otherwise mentioned. Sample size is not constant due to missing values in some variables: All – Marital status (*n =* 4132), Education level (*n =* 3735), Body mass index (*n =* 3735), Smoking habits (*n =* 4130), Alcohol drinking (*n =* 4129); 60-64y – Marital status (*n = 965*), Education level (*n = 965*), Body mass index (*n = 920*), Smoking habits (*n = 965*), Alcohol drinking (*n = 964*); 65-69y – Marital status (*n = 941*), Education level (*n = 943*), Body mass index (*n = 900*); 70-74y –Education level (*n = 813*), Body mass index (*n = 744*), Smoking habits (*n = 818*); 75-79y –Education level (*n = 735*), Body mass index (*n = 644*); 80-84y –Education level (*n = 432*), Body mass index (*n = 361*), Smoking habits (*n = 435*), Alcohol drinking (*n = 435*); 85 + y – Education level (*n = 232*), Body mass index (*n = 166*), Smoking habits (*n = 232*), Alcohol drinking (*n = 231*); NUTS II, Nomenclature of territorial units for statistics II; SD, standard deviation

Ageing trajectories for continuous outcomes (disability index, HRQoL) were estimated with linear mixed models, considering age as a fixed effect and individuals as the random intercept. These linear trajectories were then plotted by baseline age group (60-64y, 65-69y, 70-74y, 75-79y, 80-84y, 85 + y) and sex. For physical function domains, the proportion of people with at least some difficulty was represented across age. The associated factors to HRQoL were assessed considering a single linear model for participants 60 + years-old following a selection in two steps. Univariate models were computed first to test the significance of potential predictors, with p < 0.25 as the selection criterion. Significant variables were then subsequently added and kept in the multivariate model if they achieved statistical significance of p < 0.05. Models were compared through likelihood ratio tests until the fully adjusted model was reached. The disability index showed non-normality of residuals when a linear model was applied. Therefore, associated factors were estimated through a left-censored tobit regression model with a specified random intercept. Body mass index’s underweight and normal weight categories were combined in the models due to sample size constraints.

All analysis and plots were computed using R Software version 4.1.1 [7], linear mixed models were computed using the *lmer* function from the *lme4* package [8] and the tobit model computed using the *censReg* package [9].

## Results

The analytic sample consisted of 4135 participants with a mean age of 71.7 ± 7.8 years (Fig. 1). The majority of participants were women (63.5%), lived in the North (28.2%) and were married or were in a partnership (60.3%). Most participants had 4 to 9 years of full-time education (59.8%) and most participants had a BMI between 25 and 29.9 kg/m^2^ (42.5%). More than two-thirds never smoked (76.7%) and close to half reported alcohol intake (either daily or occasionally) (49.6%) – Table 1, e-Table 1. Participants were followed for a mean period of 3.6 ± 3.4 years with a maximum of 9.8 years.

### Physical function (disability index and domains)

**Fig. 2:**
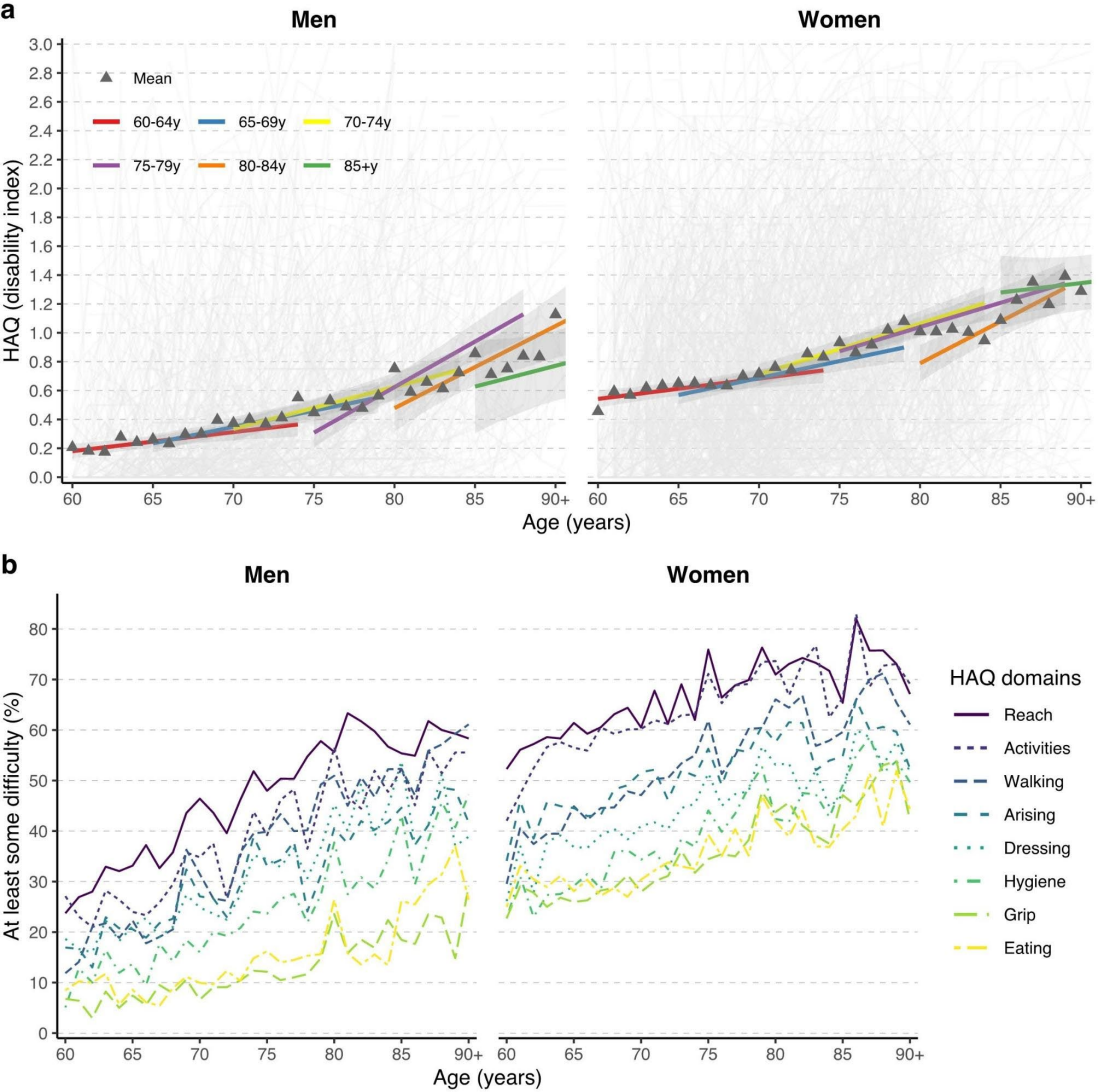
Trajectories of disability for the disability index (**a**) of the health assessment questionnaire (HAQ) in older adults by baseline age group over 10 years. Triangles are the means for each age and are not the individual points used for plotting the trajectories. HAQ, health assessment questionnaire. Models sample size: Men – 60–64 (*n =* 865), 65–69 (*n =* 817), 70–74 (*n =* 668), 75–79 (*n =* 566), 80–84 (*n =* 275), 85+ (*n =* 112); Women – 60–64 (*n =* 339), 65–69 (*n =* 324), 70–74 (*n =* 259), 75–79 (*n =* 210), 80–84 (*n =* 93), 85+ (*n =* 40). Trajectories of the percentage of people with at least some difficulty in disability domains (**b**) of the health assessment questionnaire by years of age over 10 years

At baseline, 24.8% of older Portuguese adults reported a lot of difficulty or were unable to perform activities in the same domains (dressing, arising, eating, and bathing or showering). On average, physical disability of Portuguese older adults increased with age (Fig. 2a), resulting in an increase in 0.26 (0.24, 0.29) points over 10 years (e-Table 2). For example, at baseline on average, those aged 60–64 had a disability index of 0.40 ± 0.58 while those aged 70–74 had an index of 0.66 ± 0.70. Older men and women also had a faster increase of disability. For example, those aged 60–64 reported an increase of 0.014 points per year in the disability index, those aged 70-74y a 0.033-point increase per year, and those 80-84y a 0.059 point increase per year (e-Table 2). This would correspond to an increase of 0.14, 0.33 and 0.56 over 10 years, respectively. Combining age groups 80-84y and 85 + y into an 80 + y group shows a statistically significant increase in disability with each year by 0.0455 points (e-Table 2).

Overall, women had higher disability than men at baseline, but the rate of increase was similar between sexes (Fig. 2a, e-Table 2). For example, women aged 65–69 had, on average, a disability index of 0.62 ± 0.66 at baseline and were expected to increase to 0.76 by the time they were 75-79y. Men of the same age had, on average, a disability index of 0.29 ± 0.52 at baseline and were expected to increase to 0.52 over 10 years.

Similarly, grouping the disability index into domains showed that the proportion of men and women who had at least some difficulty in one of the activities of daily living (dressing, arising, eating, walking, hygiene, reach, grip and activities) increased with age, albeit with some fluctuations), e.g., 12%, 31%, 51% of men and 30%, 47%, 66% of women at 60, 70 and 80 years old, respectively, had at least one difficulty in the walking domain. In particular, reach and common activities (e.g., run errands and shop) were the domains where most people reported difficulties in, e.g., 24% and 52% of men and women aged 60y, respectively, and 58% of men and 67% of women aged 90+, respectively, reported having at least one difficulty reaching. Grip and eating domains seemed to be better conserved, ranging from only 3% in younger to 37% in older men, and 23% in younger to 54% in older women (Fig. 2b). Overall, there were more women than men with at least some difficulty in any domain at most ages, but women had difficulties in similar domains as men. However, there were slight differences, such as that for women, dressing does not appear to be as difficult as walking or arising while for men these domains show similar degrees of difficulty.

Being older, being a woman, having multimorbidity, and being overweight or obese was associated with a higher score in the disability index or lower physical function. Higher education, exercising frequently, and being an alcohol drinker (any quantity vs. non-drinkers) were associated with a lower score in the disability index or higher physical function (Table 2).

**Table 2:** Estimates and 95% confidence intervals (95% CIs) of the factors associated with physical function (HAQ disability index)

	$\varvec{\beta }$	95% CI
**Age** (years)	0.031	(0.027, 0.035)
**Sex**		
Men	ref.	ref.
Women	0.461	(0.398, 0.523)
**Education** (years)	-0.038	(-0.047, -0.029)
**Exercise**		
Non-frequent	ref.	ref.
Frequent	-0.077	(-0.155, 0.000)
Very frequent	-0.165	(-0.216, -0.114)
**Alcohol drinking**		
No	ref.	ref.
Yes	-0.083	(-0.126, -0.041)
**Multimorbidity** (yes)	0.446	(0.384, 0.507)
**Body mass index** (kg/m^2^)		
Underweight/Normal weight (< 25)	ref.	ref.
Overweight (25-29.9)	0.081	(0.025, 0.137)
Obese (≥ 30)	0.232	(0.162, 0.301)

HAQ: health assessment questionnaire; $\beta$: coefficient estimates; ref: reference class; multivariate model adjusted for age, sex, education, exercise, alcohol, multimorbidity and body mass index; model sample size (*n =* 2840)

### Health-related quality of life

**Fig. 3:**
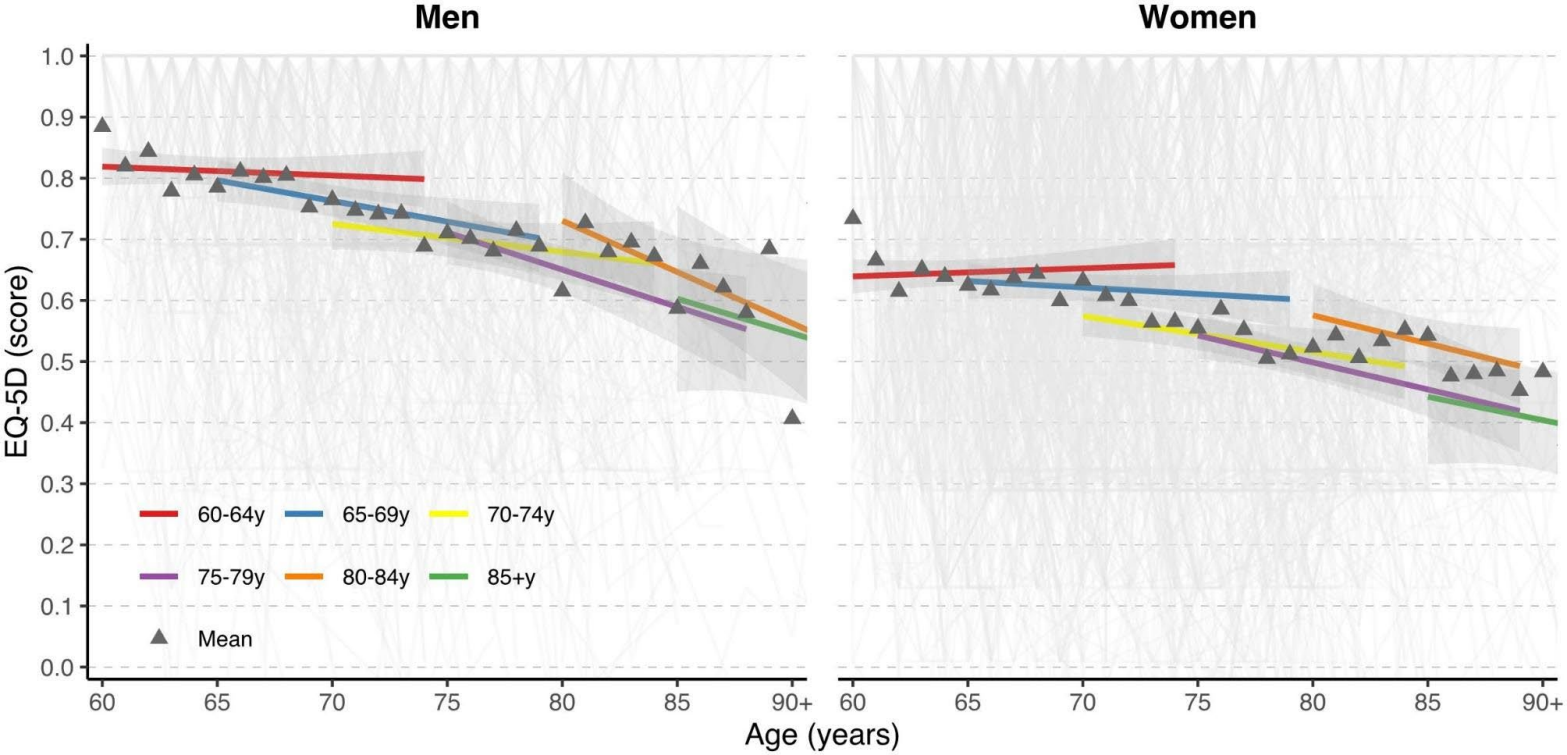
Trajectories of health-related quality of life (EQ-5D score) in older adults by baseline age group over 10 years. Triangles are the means for each age and are not the individual points used for plotting the trajectories. EQ-5D, EuroQol 5-dimensions. Models sample size: Men – 60–64 (*n =* 280), 65–69 (*n =* 251), 70–74 (*n =* 213), 75–79 (*n =* 166), 80–84 (*n =* 73), 85+ (*n =* 28); Women – 60–64 (*n =* 471), 65–69 (*n =* 454), 70–74 (*n =* 371), 75–79 (*n =* 325), 80–84 (*n =* 159), 85+ (*n =* 60)

On average, the HRQoL of Portuguese older adults declined by 0.074 (-0.084, -0.063), over 10 years. There was a general decrease in the mean HRQoL of older adults across age (Fig. 3, e-Table 2). For example, baseline 60–64 year-olds had and average HRQoL score of 0.76 ± 0.25, decreasing to 0.69 ± 0.29 for 70-74y and 0.60 ± 0.31 at 80-85y. This decrease was generally higher the older the baseline age group, e.g., 65–69 year-olds had a decrease of HRQoL of 0.004 points each year, 70–74 year-olds decreased by 0.005 points each year, 75–79 year-olds decreased by 0.010 points, and 80–84 year-olds decreased by 0.012 points each year (Fig. 3, e-Table 2). This is the equivalent of 0.04, 0.05, 0.10 and 0.12 points over the course of 10 years, respectively. When participants from age groups 80-84y and 85 + y are combined into an 80 + y group, we see that their HRQoL significantly decreases with age by 0.0135 points each year (e-Table 2).

On average, women tended to have lower HRQoL than men for all age groups but with an overall slower decrease across most age groups (Fig. 3, e-Table 2). For example, men aged 65–69 years declined by 0.007 points per year while women of the same age declined 0.002 points per year. The highest decrease found was for men in the 80-84y range, worsening their HRQoL 0.17 points over 10 years.

Similarly, to physical function, being older, being a woman, having multimorbidity, and being obese were associated with lower HRQoL. Higher education, frequent exercise and being an alcohol drinker (any quantity vs. non-drinkers) were associated with higher HRQoL (Table 3).

**Table 3:** Estimates and 95% confidence intervals (95% CIs) of the factors associated with health-related quality of life

	$\varvec{\beta }$	95% CI
**Age** (years)	-0.007	(-0.008, -0.006)
**Sex**		
Men	ref.	ref.
Women	-0.097	(-0.1170, -0.078)
**Education** (years)	0.012	(0.0092, 0.015)
**Exercise**		
Non-frequent	ref.	ref.
Frequent	0.054	(0.027, 0.082)
Very frequent	0.071	(0.052, 0.090)
**Alcohol drinking**		
No	ref.	ref.
Yes	0.039	(0.024, 0.054)
**Multimorbidity** (yes)	-0.131	(-0.151, -0.110)
**Body mass index** (kg/m^2^)		
Underweight/Normal weight (< 25)	ref.	ref.
Overweight (25-29.9)	-0.007	(-0.026, 0.012)
Obese (≥ 30)	-0.048	(-0.071, -0.025)

$\beta$: coefficient estimates; ref: reference class; multivariate model adjusted for age, sex, education, exercise, alcohol, multimorbidity and body mass index; model sample size (*n =* 2467)

## Discussion

To the best of our knowledge, this is the first study to offer a broad picture of the ageing trajectories of older adults in Portugal over 10 years. The study examined the progression of HRQoL and physical function of more than 4100 adults aged 60 + over the course of 10 years (mean follow-up 3.6 ± 3.4 years). With advancing age, older adults reported reduced HRQoL and increased physical disability. Moreover, decline in health tended to be faster for older age groups. Women were, in general, in worse health at baseline than men but the rate of change was similar. Regarding associated factors, results suggest that higher education and practicing regular exercise may be protective for physical function and HRQoL while having multimorbidity and excess weight seem to be detrimental.

Decline in health was faster for older age groups up until 80-84y. We believe these are the effects of having a smaller sample size in the age group 85 + y, since when looking at the combined 80 + y group, we see a faster decline in HRQoL from the 75-79y group.

In our study, women had a higher disability index and more had multimorbidity than men at baseline but the rate of change was similar between sexes, which is in line with most ageing studies [10, 11]. These findings are probably partly due to survival bias as men who have been selected and were able to participate in the study were in better health than women who have, on average, higher life expectancies. Studies with middle-aged adults do not report a similar gender paradox and have found that morbidity is not consistently higher in women than in men [12]. Furthermore, increased physical disability with age is closely related with reduced HRQoL [13]. These results are in line with data from Ireland, Scotland and the Study on global AGEing and adult health, where HRQoL decreased with age in older adults, markedly more so in women [14–16]. This decrease in HRQoL in later years was associated with lower education, higher food insecurity, loneliness, poverty, and mental health [14, 15, 17].

We found that reach and common activities (e.g., running errands, shopping, cleaning the house) were the domains where participants had, overall, the most difficulty with and, grip and eating were the domains where the least difficulty was observed. Similarly, a study with a population from Newcastle and North Tyneside found that very old adults (85 years and older) had more difficulty shopping, using steps, walking and washing all over, and less difficulty eating, transferring from bed and dressing, with women also having a higher prevalence of difficulty in all items relative to men [18]. Previous longitudinal studies have also reported an increase in the difficulty performing activities of daily living with older age, particularly an increased difficulty in eating, in walking, bathing and transferring [19–21]. Across all EU member states (2013-15) 8.4% of older adults reported having a lot of difficulty performing at least one of five activities of daily living (eating and drinking, getting in or out of a bed or chair, dressing and undressing, using the toilet, and bathing or showering) with Portugal being slightly above the European average [22]. In this study, 24.8% of older Portuguese adults reported a lot of difficulty or were unable to perform activities in the same domains (dressing, arising, eating, and bathing or showering).

Being an alcohol drinker vs. not drinking showed an apparent beneficial effect on physical function and HRQoL of older adults. This effect is predominant in our cohort across a variety of subpopulations, even in younger ages [23, 24]. The most likely explanation is that alcohol drinking’s (vs. non-drinkers) positive effects are largely due to reverse causation and the “sick quitter” hypothesis, which states that a considerable proportion of those that do not drink quit drinking in the past for health reasons [25]. Another much less likely explanation is that although excessive alcohol consumption is associated with worse HRQoL and physical function [26], higher rates of moderate alcohol consumption may also be associated with higher socioeconomic status [27], which in turn is a determinant of HRQoL and physical function.

An increase in multimorbidity comes with a huge cost for the HRQoL of older adults, and for healthcare systems worldwide [28]. Previously, we showed that Portuguese older adults had a high prevalence of multimorbidity (78%), which increased across age strata, and unhealthy lifestyles [3]. In the present study, physical disability increased with age and the rate of change was, generally, faster in those who were older, which is also when the prevalence of multimorbidity is the highest. Apart from the ageing process and its consequences, Portuguese older adults have poor socioeconomic conditions and unhealthier lifestyles which make them an especially vulnerable group to multimorbidity [3]. Low education level and obesity have been linked with multimorbidity. In our sample, a third of older adults had only four or fewer years of education with the majority having 5–9 years, and over 60% were overweight or obese [29].

Physical activity, including regular exercise practice, is extensively reported to have health benefits. Importantly, it is associated with lower risk for all-cause mortality, cardiovascular diseases and cancer [30–32], as well as improved HRQoL [33]. With age, particularly after 60 the proportion of people that perform regular exercise tends to decrease [34]. Furthermore, despite the COVID-19 pandemic lockdowns from March to August 2021, which affected the usual exercise routines of people who were physically active before, and also the fact that participants were much older at the end of the study, there was an overall beneficial effect of exercise over the 10 years of follow-up.

These estimations may help the Portuguese government and other decision-makers to effectively plan budget and human resource allocation to long term care and healthcare, targeting those that need it the most, as well as ensuring availability of carers and supporting informal carers.

### Strengths and limitations

This study has important limitations and strengths that should be taken into consideration when interpreting the results. As all data were self-reported, the probability of recall bias was high. However, for outcomes such as HRQoL, participants were asked about the current health status and were, therefore, less prone to recall bias. Over 60% of the Portuguese population have a low level of health literacy which may have resulted in misclassification of the self-reported number of chronic diseases diagnosed [35]. There were slight differences between questionnaires in each wave that had to be harmonized, e.g., previous (any period) diagnosis of chronic diseases was asked at baseline and wave 4, but for waves 2 and 3 it was asked as diagnosis since the previous contact. Similarly, a more informative definition of regular exercise frequency could not be derived.

It is important to note that the estimations for the prevalence and rate of progression of HRQoL and physical function are, in all likelihood, small to moderate underestimations since there was a 30, 26 and 43% of dropout between EpiDoC 1 and 2, EpiDoC 2 and 3, and EpiDoC 3 and 4, respectively. A more detailed collection of the dropout reasons would be important to further quantify the follow-up bias. For example, mortality is essential to accurately determine the trajectories of older adults and the causes of dropout. However, because mortality was only ascertained through successful contacts with a family member/acquaintance and no linkage with the national mortality database was possible, the number (and timing) of deaths was severely underestimated and not included in the analysis. Stratification of results by sex and age group limited sample size, especially in the 85 + y where sample size was below 100 individuals. However, results in an 80 + y group provided robust estimates in line with the expected aggravating effects of age. We also note that due to limited sample size, analysis was not stratified by NUTS II region, and only a general description was presented.

A considerable strength is that the EpiDoC cohort was representative of the adult Portuguese population with over 4100 older adults who were followed up to 10 years. Moreover, all questionnaires were validated for the Portuguese population which better captures the health perceptions.

## Conclusions

Using data from a large population-based prospective cohort study we were able to estimate the health trajectories of Portuguese older adults over 10 years and evaluate associated factors with physical function and HRQoL. With advancing age, older adults reported increased physical disability and reduced health-related quality of life. Higher education and regular exercise seemed to be protective for physical function and HRQoL, while multimorbidity and excess weight were associated with poor physical function and HRQoL over time. These findings are essential to effectively plan resource allocation, plan better healthcare and design informed public health policies focusing on health promotion and bridging the gap between life expectancy and healthy life expectancy in Portugal.

### Electronic supplementary material

Below is the link to the electronic supplementary material.

Supplementary Material 1

## Data Availability

The codebook and analytic code are available pending request from the authors while the dataset is available pending application and approval by the EpiDoC Coordinator - Ana Rodrigues (ana.m.rodrigues@nms.unl.pt).
