# Supplementary material for: Trajectories and determinants of ageing in Portugal: insights from EpiDoC, a nationwide population-based cohort
Source: BMC Public Health. 2023 Aug 17;23:1564. doi: 10.1186/s12889-023-16370-8 (PMC10433601; doi:10.1186/s12889-023-16370-8)
Supplement: Supplementary file 1 — Supplementary Material 1 [file 12889_2023_16370_MOESM1_ESM.docx]

**Supplementary material**

**e-Table 1.** Baseline sociodemographic and lifestyle characteristics of older adults from the EpiDoC cohort.

|  | **All**  *n=*4135 | **Men**  *n=*1510 (36.5%) | **Women**  *n=*2625 (63.5%) |
| --- | --- | --- | --- |
| **Sociodemographic** |  |  |  |
| **Age** (years) |  |  |  |
| Mean (SD) | 71.7 (7.8) | 71.4 (7.7) | 72.0 (7.8) |
| 60-64 | 966 (23.4%) | 372 (24.6%) | 594 (22.6%) |
| 65-69 | 943 (22.8%) | 359 (23.8%) | 584 (22.2%) |
| 70-74 | 819 (19.8%) | 300 (19.9%) | 519 (19.8%) |
| 75-79 | 737 (17.8%) | 260 (17.2%) | 477 (18.2%) |
| 80-84 | 437 (10.6%) | 140 (9.3%) | 297 (11.3%) |
| ≥85 | 233 (5.6%) | 79 (5.2%) | 154 (5.9%) |
| **Region** (NUTS II) |  |  |  |
| North | 1168 (28.2%) | 465 (30.8%) | 703 (26.8%) |
| Centre | 898 (21.7%) | 361 (23.9%) | 537 (20.5%) |
| Lisbon | 869 (21.0%) | 302 (20.0%) | 567 (21.6%) |
| Alentejo | 319 (7.7%) | 106 (7.0%) | 213 (8.1%) |
| Algarve | 165 (4.0%) | 53 (3.5%) | 112 (4.3%) |
| Azores | 328 (7.9%) | 120 (7.9%) | 208 (7.9%) |
| Madeira | 388 (9.4%) | 103 (6.8%) | 285 (10.9%) |
| **Marital status** |  |  |  |
| Single | 190 (4.6%) | 38 (2.5%) | 152 (5.8%) |
| Married/Partnership | 2491 (60.3%) | 1217 (80.7%) | 1274 (48.6%) |
| Divorced | 211 (5.1%) | 140 (9.3%) | 149 (5.7%) |
| Widowed | 1240 (30.0%) | 191 (12.7%) | 1049 (40.0%) |
| **Education level** |  |  |  |
| <4 years | 1201 (29.2%) | 249 (16.5%) | 952 (36.5%) |
| 4-9 years | 2460 (59.8%) | 1058 (70.2%) | 1058 (70.2%) |
| 10-12 years | 229 (5.6%) | 109 (7.2%) | 120 (4.6%) |
| >12 years | 226 (5.5%) | 91 (6.0%) | 135 (5.2%) |
| **Lifestyle** |  |  |  |
| **Body mass index**(kg/m^2^) |  |  |  |
| Mean (SD) | 27.6 (4.6) | 27.3 (4.0) | 27.8 (4.9) |
| Underweight (<18.5) | 38 (1.0%) | 9 (0.6%) | 29 (1.3%) |
| Normal weight (18.5-24.9) | 1114 (29.8%) | 450 (31.1%) | 664 (29.0%) |
| Overweight (25-29.9) | 1589 (42.5%) | 646 (44.6%) | 943 (41.2%) |
| Obese (≥30) | 994 (26.6%) | 343 (23.7%) | 651 (28.5%) |
| **Smoking habits** |  |  |  |
| Never | 2980 (72.2%) | 568 (37.7%) | 2412 (92.0%) |
| In the past | 878 (21.3%) | 735 (48.7%) | 143 (5.5%) |
| Active smokers | 272 (6.6%) | 205 (13.6%) | 67 (2.6%) |
| **Alcohol drinking** |  |  |  |
| No | 2803 (50.4%) | 363 (24.1%) | 1720 (65.6%) |
| Yes | 2046 (49.6%) | 1144 (75.9%) | 902 (34.4%) |

All values are n (%) unless otherwise mentioned. Sample size is not constant due to missing values in some variables: All – Marital status (*n=*4132), Education level (*n=*3735), Body mass index (*n=*3735), Smoking habits (*n=*4130), Alcohol drinking (*n=*4129); Men – Marital status (*n=*1508), Education level (*n=*1507), Body mass index (*n=*1448), Smoking habits (*n=*1508), Alcohol drinking (*n=*1507); Women – Marital status (*n=*2624), Education level (*n=*2609), Body mass index (*n=*2287), Smoking habits (*n=*2622), Alcohol drinking (*n=*2622). NUTS II, Nomenclature of territorial units for statistics II; SD, standard deviation.

**e-Table 2**. Health-related quality of life (HRQoL) and physical function’s linear mixed model coefficients for the fixed effect of age (years).

|  |  | **Age** | |
| --- | --- | --- | --- |
|  | n | $\beta$ | 95% CI |
| **Physical function**  (HAQ) |  |  |  |
|  |  |  |  |
| **Total** |  |  |  |
| 60+ | 3303 | 0.0263 | (0.0240, 0.0286) |
|  |  |  |  |
| **Total** |  |  |  |
| 60-64 | 865 | 0.0138 | (0.0081, 0.0194) |
| 65-69 | 817 | 0.0238 | (0.0173, 0.0303) |
| 70-74 | 668 | 0.0332 | (0.0254, 0.0410) |
| 75-79 | 566 | 0.0447 | (0.0342, 0.0553) |
| 80-84 | 275 | 0.0588 | (0.0417, 0.0757) |
| 80+ | 387 | 0.0439 | (0.0316, 0.0562) |
| 85+ | 112 | 0.0196 | (-0.0070, 0.0460) |
|  |  |  |  |
| **Men** |  |  |  |
| 60-64 | 339 | 0.0132 | (0.0055, 0.0210) |
| 65-69 | 324 | 0.0227 | (0.0129, 0.0325) |
| 70-74 | 259 | 0.0291 | (0.0165, 0.0417) |
| 75-79 | 210 | 0.0631 | (0.0460, 0.8010) |
| 80-84 | 93 | 0.0569 | (0.0271, 0.0860) |
| 80+ | 133 | 0.0388 | (0.0188, 0.0586) |
| 85+ | 40 | 0.0288 | (0.0121, 0.0696) |
|  |  |  |  |
| **Women** |  |  |  |
| 60-64 | 526 | 0.0142 | (0.0065, 0.0218) |
| 65-69 | 493 | 0.0235 | (0.0151, 0.0319) |
| 70-74 | 409 | 0.0353 | (0.0254, 0.0452) |
| 75-79 | 356 | 0.0336 | (0.0205, 0.0467) |
| 80-84 | 182 | 0.0580 | (0.0371, 0.0789) |
| 80+ | 254 | 0.0455 | (0.0302, 0.0607) |
| 85+ | 72 | 0.0130 | (-0.0202, 0.0461) |
|  |  |  |  |
|  |  |  |  |
| **HRQoL**  (EQ-5D) |  |  |  |
|  |  |  |  |
| **Total** |  |  |  |
| 60+ | 2851 | -0.0074 | (-0.0084, -0.0063) |
|  |  |  |  |
| **Total** |  |  |  |
| 60-64 | 751 | 0.0004 | (-0.0026, 0.0034) |
| 65-69 | 705 | -0.0039 | (-0.0073, -0.0005) |
| 70-74 | 584 | -0.0053 | (-0.0092, -0.0014) |
| 75-79 | 491 | -0.0104 | (-0.0154, -0.0052) |
| 80-84 | 232 | -0.0120 | (-0.0199, -0.0041) |
| 80+ | 320 | -0.0135 | (-0.0190, -0.0080) |
| 85+ | 88 | -0.0083 | (-0.0205, 0.0040) |
|  |  |  |  |
| **Men** |  |  |  |
| 60-64 | 280 | -0.0014 | (-0.0058, 0.0029) |
| 65-69 | 251 | -0.0067 | (-0.0120, -0.0015) |
| 70-74 | 213 | -0.0046 | (-0.0109, 0.0018) |
| 75-79 | 166 | -0.0122 | (-0.0209, -0.0033) |
| 80-84 | 73 | -0.0168 | (-0.0319, -0.0012) |
| 80+ | 101 | -0.0167 | (-0.0262, -0.0072) |
| 85+ | 28 | -0.0113 | (-0.0304, 0.0079) |
|  |  |  |  |
| **Women** |  |  |  |
| 60-64 | 471 | 0.0013 | (-0.0027, 0.0053) |
| 65-69 | 454 | -0.0021 | (-0.0065, 0.0023) |
| 70-74 | 371 | -0.0059 | (-0.0108, -0.0010) |
| 75-79 | 325 | -0.0088 | (-0.0150, -0.0026) |
| 80-84 | 159 | -0.0092 | (-0.0183, -0.0001) |
| 80+ | 219 | -0.0119 | (-0.0186, -0.0052) |
| 85+ | 60 | -0.0077 | (-0.0232, 0.0080) |
|  |  |  |  |

$\beta$ – beta coefficient, 95% CI – 95% confidence interval; models adjusted for age.

**e-Figure 1.** Trajectories of disability (HAQ) in older adults (both men and women) by baseline age group over 10 years. Triangles are the means for each age and are not the individual points used for plotting the trajectories. HAQ, health assessment questionnaire.

**e-Figure 2.** Trajectories of disability domains (HAQ) in older adults (both men and women) over 10 years. HAQ, health assessment questionnaire.

**e-Figure 3.** Trajectories of health-related quality of life (EQ-5D score) in older adults (both men and women) by baseline age group over 10 years. Triangles are the means for each age and are not the individual points used for plotting the trajectories. EQ-5D, EuroQol 5-dimensions.
